# Enhanced Fault Detection in Photovoltaic Panels Using CNN-Based Classification with PyQt5 Implementation

**DOI:** 10.3390/s24227407

**Published:** 2024-11-20

**Authors:** Younes Ledmaoui, Adila El Maghraoui, Mohamed El Aroussi, Rachid Saadane

**Affiliations:** 1 Laboratory Engineering System, Hassania School of Public Works, Casablanca BP 8108, Morocco; 2 Green Tech Institute, Mohammed VI Polytechnic University, Benguerir BP 43150, Morocco

**Keywords:** solar energy, artificial intelligence, fault detection, sustainability, solar panel, renewable energy, predictive maintenance

## Abstract

Solar photovoltaic systems have increasingly become essential for harvesting renewable energy. However, as these systems grow in prevalence, the issue of the end of life of modules is also increasing. Regular maintenance and inspection are vital to extend the lifespan of these systems, minimize energy losses, and protect the environment. This paper presents an innovative explainable AI model for detecting anomalies in solar photovoltaic panels using an enhanced convolutional neural network (CNN) and the VGG16 architecture. The model effectively identifies physical and electrical changes, such as dust and bird droppings, and is implemented using the PyQt5 Python tool to create a user-friendly interface that facilitates decision-making for users. Key processes included dataset balancing through oversampling and data augmentation to expand the dataset. The model achieved impressive performance metrics: 91.46% accuracy, 98.29% specificity, and an F1 score of 91.67%. Overall, it enhances power generation efficiency and prolongs the lifespan of photovoltaic systems, while minimizing environmental risks.

## 1. Introduction

The reliance on fossil fuels for electricity generation has become a significant contributor to greenhouse gas emissions (GHGs) [1], leading to detrimental effects on the environment, such as climate change and air pollution. In stark contrast, the adoption of renewable energy sources, especially solar power, offers a pathway to effectively mitigate these impacts. According to recent reports, the global renewable energy capacity saw a remarkable increase of 257 GW in 2021, reaching a total of 3064 GW. Among energy sources, solar energy emerged as the frontrunner, with an increase of 133 GW, marking a 19% growth and pushing the global solar capacity to 849 GW [2].

**Figure 1:**
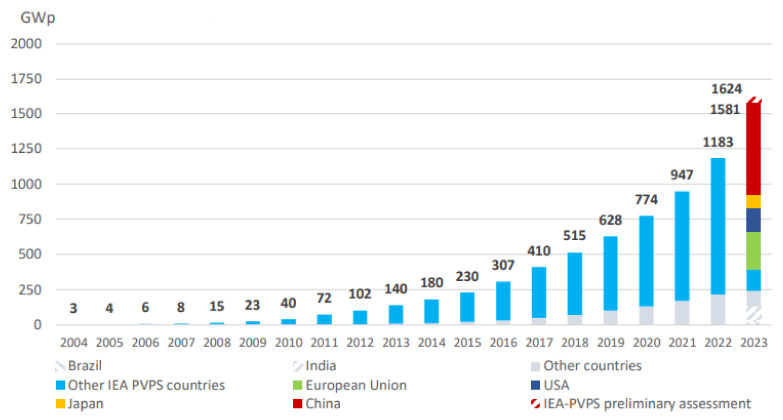
Evolution of installed solar capacity from 2004 to 2023 [4].

China played a pivotal role in this growth, enhancing its solar capacity from 253 GW to 307 GW, making it the largest contributor globally. The United States also made significant strides, boosting its solar output by 94 GW [3], which represents a 27% increase. This expansion has shifted the Asia–Pacific region into a leading position, now accounting for over 60% of the world’s photovoltaic (PV) installations, totaling at least 947 GW. This trend of increasing solar energy adoption reflects the growing recognition of its potential to provide sustainable energy solutions, as depicted in Figure 1.

Solar energy not only reduces GHG emissions but also promotes energy security and economic growth through job creation in the renewable energy sector. Additionally, with technological advancements in solar panel efficiency and energy storage solutions, the future looks promising for solar power to play a central role in global energy strategies.

**Figure 2:**
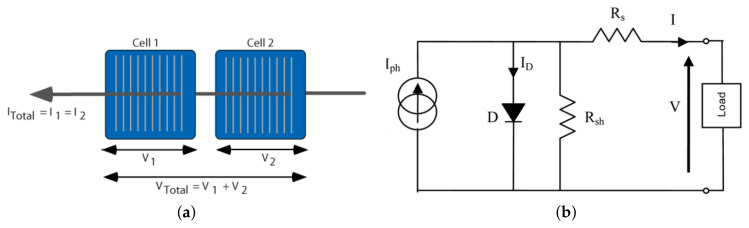
PV cell (**a**), electrical schematic diagram (**b**).

**Figure 3:**
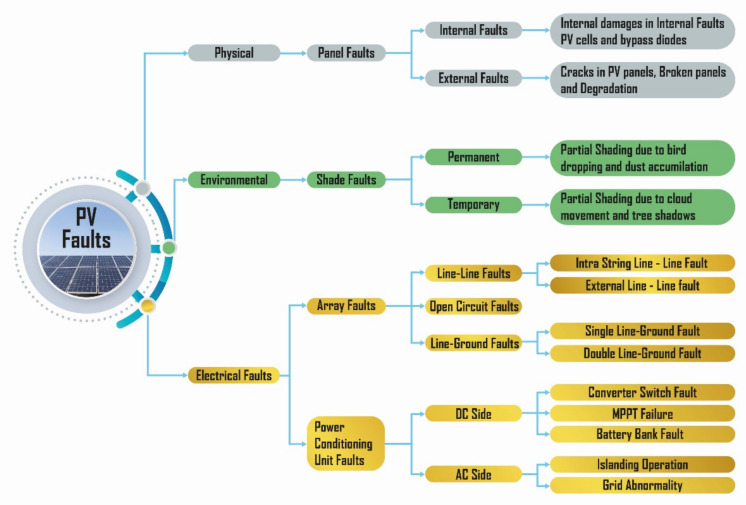
PV system fault classification.

Photovoltaic (PV) cells, depicted in Figure 2, are a solar technology that converts solar energy into electricity with a nominal efficiency ranging from 15% to 20% [5]. This efficiency, however, affects the global adoption rate of solar energy [6], as the maximum performance of PV systems depends on several environmental factors, as shown in Figure 3. These include the accumulation of dust on the PV surface, operating temperature, hail, snow, wind speed, shading, air density, and sky conditions. Among these factors, soiling losses due to dust, dirt, and other particles are particularly detrimental to PV module performance.

Dust refers to any particle less than 10 mm in diameter and originating from various sources such as sand, dirt, construction debris, rocks, volcanic ash, bird droppings, and eroded limestone [7]. While dust accumulation can lead to reduced energy generation, it can also exacerbate soiling effects on panels [8]. Factors such as ambient temperature, tilt angle, soil conditions, and nearby vegetation significantly influence dust deposition, along with the cover material of the PV module and the angle of sunlight [9]. Dust can be deposited in three distinct ways: occult (mist, clouds, high humidity), wet (rainfall), and dry (wind). The composition of dust varies based on local environmental conditions, with higher deposition rates occurring near industrial areas, in volcanic regions, and in areas prone to sandstorms [10].

### 1.1. Different PV System Faults

PV systems experience a wide range of problems from being located outdoors, which can significantly lower the PV energy output, reduce the potential, and most importantly make it impossible to meet different load demands. Three basic categories can be used to classify faults: physical, environmental, and electrical, as summarized in Figure 3.

Electrical faults include short circuits, circuit breaks, bypass diode faults, and shunt resistance insertion faults. These reduce the voltage and current, decreasing the power output. Environmental faults include shading, which usually causes the bypass effect, where the corresponding currents are shifted and lower rated than the performance could be at that point. Physical faults include micro-cracks and internal damages.

PV modules’ performance and efficiency vary based on the dust accumulation level in their surrounding environment [11]. For example, dust particles significantly reduce irradiation levels, scattering the wavelengths of the incoming radiation. When a thick layer of dust coats a module’s surface, it alters its optical properties, increasing light reflection and decreasing transmissivity, which ultimately leads to a decrease in electrical output. Additionally, dust accumulation can increase temperatures, causing a slight reduction in open-circuit voltage (by 2–6%) and short-circuit current (by 15–20%). Research has shown that a dust level of 4.25 mg/cm² can reduce the output power by 33%, while dusty modules produced 8.41% less power compared to clean ones.

Artificial intelligence (AI) can assist in prioritizing maintenance tasks and optimizing the scheduling of inspections and repairs by recognizing patterns and trends. The proposed AI-based detection system addresses this challenge by using deep learning models to automatically identify and classify faults from image data. This approach offers an efficient, cost-effective, and scalable solution for real-time monitoring, ultimately enhancing the performance and reliability of solar energy systems and supporting the growth of renewable energy infrastructure.

### 1.2. Contributions and Limitations

The main contributions of this paper are as follows:

+ Exploring advancements and integrations of AI in solar panel systems.
+ Addressing challenges in detecting and classifying anomalies, which is crucial for optimizing performance.
+ Identifying and classifying the factors contributing to anomalies in PV power, which is essential for evaluating their impact on model accuracy.
+ Implementing a model tested with PyQt5, enhancing user decision-making by providing an intuitive interface that simplifies interaction with the solar energy system.

While the proposed AI-based detection system significantly contributes to enhancing solar panel system performance, there are some limitations associated with the objectives of this research:

- The reliance on image-based data introduces challenges related to environmental factors, such as lighting.
- The model’s ability to classify rare or unseen anomalies is limited by the diversity of fault data used in training, potentially reducing the accuracy in real-world applications.

Despite these limitations, the model provides a solid foundation for further advancements in AI-driven anomaly detection in solar systems.

The remainder of the paper is structured as follows. The related works are discussed in Section 2. The methodology is presented in Section 3. Section 4 discusses the obtained results. The conclusions based on the outcome of the analysis phase are presented in Section 5.

## 2. Related Works

The advancement of artificial intelligence (AI)-powered dust detection systems has been the focus of researchers [12]. Various methods, such as k-nearest neighbors (kNN) and random forest, have been utilized to classify and detect dusty panels, along with the application of deep learning models for this purpose [13].

Analytical measurement of solar panel performance can be performed using a LDR and multi-meter [14]. Using a single hidden layer containing nine neurons, an artificial neural network was established to predict the output voltage of solar panels based on input metrics like irradiance and dust content. Additionally, a deep residual neural network and image processing were employed to forecast uneven dust accumulation [15].

The CNN LeNet model with customized dropouts and pooling layers was used to achieve a mean squared error of 0.0122 and an 80% accuracy. Deep CNN architectures were also used to develop a model from a dataset of 599 photos, achieving a 93.3% accuracy with the AlexNet model. The authors studied PV deterioration and irregularity patterns using various machine learning and deep learning methods, considering the computation time, characterization techniques, datasets, and feature extraction processes [6,16,17].

### 2.1. Review of Computer Vision Applications

To broaden the scope of this research, Table 1 provides an overview of notable computer vision applications in various fields. This highlights the diversity and impact of CV technologies, positioning the proposed study within a broader context of CV advancements.

### 2.2. Prior Research in PV Fault Detection

Previous studies have primarily focused on testing models for classifying fault detection in photovoltaic systems. In one study, a deep belief network was created to identify dust on PV panels, and the suggested model outperformed previous machine-learning-based models in terms of accuracy [23]. A combination of the physical lotus effect approach with the Mobile-Net and VGG-16 CNN methodologies for the evaluation of solar panels was considered in [24]. Similarly, the performance of other machine learning algorithms, such as Facebook-prophet, isolation forest, and auto-encoder long short-term memory (AE-LSTM), was assessed for PV performance research. The outcomes provided straightforward insights to help with decision-making [25]. The impacts of dust and temperature on PV power generation were assessed using a deep-learning-based modular neural network in a subsequent study, which involved six PV modules in Sohar, Oman [26]. This paper significantly improves the CNN accuracy and performance by implementing the model within the PyQt5 framework. This advancement enables the classification of six distinct fault types, employing more advanced CNN architectures. Additionally, the integration of the model into a user-friendly PyQt5 interface enhances its accessibility and usability, making it a practical tool for real-world applications. Table 2 provides a comprehensive summary of prior research in solar panel fault detection.

## 3. Materials and Methods

### 3.1. CNN Model

The primary goal of this project is to automate the detection of anomalies in solar panels using a deep learning approach [34]. The system classifies images of solar panels into different categories based on whether they are faulty or functioning correctly. The system learns to detect and classify visual patterns from labeled solar panel images using a convolutional neural network (CNN), specifically fine-tuned from the VGG16 architecture [35]. The CNN model works by processing large datasets of solar panel images to identify unique features and patterns associated with anomalies, such as cracks, dirt, or physical damage. The trained model can accurately predict the type of anomaly or confirm that the panel is functioning normally when provided with new, unseen images [36].

### 3.2. Ensemble Learning Classifier

Ensemble learning improves machine learning results by integrating multiple models [37]. This approach involves training a group of classifiers or an ensemble, and then combining their predictions for classifying unseen examples through a voting mechanism. By leveraging this strategy, the prediction performance can surpass that of any single model. The fundamental idea is to train a diverse group of classifiers and allow them to contribute their insights. An ensemble model is developed by merging base models, addressing classification or regression challenges that individual models may struggle to solve effectively [38]. Consequently, ensemble learning can yield superior outcomes compared to using a standalone model [39].

### 3.3. Explainable Artificial Intelligence (XAI): LIME Approach

Machine learning models have often been viewed as opaque “black boxes”, but explainable artificial intelligence (XAI) techniques have emerged to clarify their functioning [40]. These methods aim to enhance users’ trust in machine learning models by providing insights into how they operate. Two widely used XAI approaches for tabular data are Shapley additive explanations (SHAP) and local interpretable model-agnostic explanations (LIME). LIME, in particular, offers local explanations that are independent of the underlying model, allowing for greater interpretability across different machine learning frameworks [41]. It illustrates how each feature influences the results for a particular instance. The classification models also indicate the probability of the instance belonging to a specified class. Moreover, it utilizes visual plots to highlight the importance of each feature within each class, enhancing the interpretability and understanding of the model’s decisions [42].

### 3.4. Solar Panel Dataset Description

**Figure 4:**
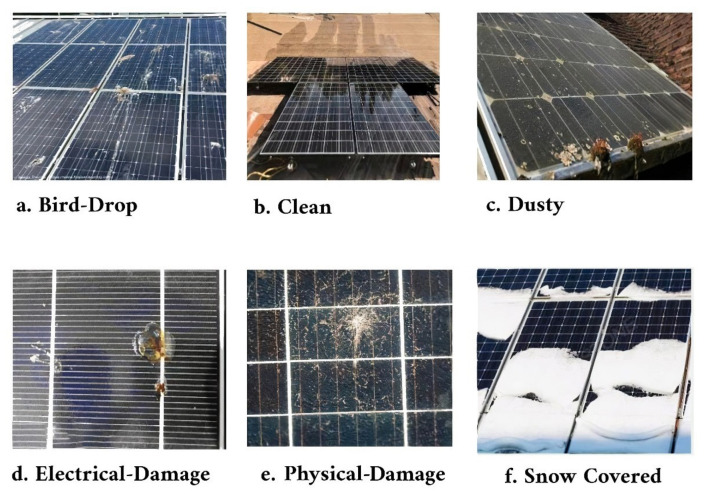
Classes of solar panels.

A dataset was designed to evaluate the performance of various ensemble machine learning classifiers and convolutional neural networks (CNNs) in detecting physical and electrical alterations to solar panel surfaces, such as dust, snow, bird droppings, and other changes. It comprises six distinct classes for classification. While the dataset is reasonably comprehensive, some imbalance exists in the number of images collected, due to their sourcing from online platforms. Table 3 provides details about the dataset, and Figure 4 illustrates examples of the six different classes present within it.

Capturing images at a moderate distance with minimal glare can help reduce noise and enable more precise pixel analysis. Optimal lighting conditions are also beneficial, as they can enhance a model’s ability to identify subtle fault indicators such as dust accumulation, physical damage, or electrical anomalies. These guidelines are intended to improve the robustness of fault detection across various environmental conditions and were incorporated to facilitate effective image capture for our model.

### 3.5. The Proposed Detection of Solar Panel Anomalies

**Figure 5:**
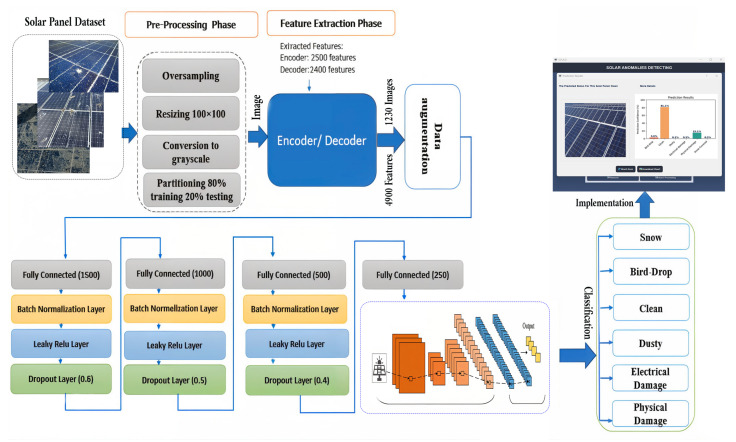
Proposed solar panel anomaly detection and classification model.

The proposed architecture consists of three key phases: preprocessing, feature extraction, and data augmentation, which generates new data points from existing ones to effectively increase the dataset size, followed by the classification phase. Figure 5 illustrates the model integrated with the ensemble classifier. Each component of the proposed model is elaborated upon in the following sections.

#### 3.5.1. Data Pre-Processing Phase

The dataset underwent oversampling to achieve a balance across all categories, resulting in each folder containing 205 images. The images were sourced from a directory housing solar panel images and were resized to 100 × 100 pixels for uniformity. To simplify the computation, RGB images were converted to grayscale. The dataset was then split into training and testing sets, allocating 80% for training and 20% for testing.

#### 3.5.2. Feature Extraction Phase

The input image is encoded into a compact knowledge representation by the autoencoder, which employs a bottleneck architecture to facilitate this compression [43]. The network employs an unsupervised learning algorithm for representation learning. The autoencoder’s core principle is to utilize its hidden layer to encode incoming sensor data, effectively creating an optimal feature representation before generating an output. This process allows the model to capture essential patterns in the data, while reducing the dimensionality. To create an output *x* that is a reconstruction of the original input *x*, an autoencoder reformulates the unlabeled dataset into a supervised learning problem. It utilizes a bottleneck structure that constrains the flow of information through the network, leading to a learned compression of the input image. This approach minimizes redundancy by focusing on the variations present in the input data. An autoencoder consists of two main components: an encoder and a decoder. The encoder transforms the input into a latent space representation using its activation function, capturing the essential features, while reducing dimensionality. This process enables effective learning and reconstruction of the original data.

#### 3.5.3. Data Augmentation Phase

To further enhance the robustness of the model, the data augmentation phase played a crucial role by creating additional training samples from the original dataset [44]. In this phase, a variety of image transformations, such as rotation, shifting, shearing, zooming, and horizontal flipping, were applied to introduce diversity to the training data. These augmentations simulated different real-world scenarios, such as varying orientations and scales of solar panels, changes in camera angles, or environmental conditions like shadows or slight misalignments. This phase not only helped the model generalize better to unseen data, making it more effective in real-world conditions, but also significantly reduced the risk of overfitting, where the model might otherwise have only learned to perform well on the training data and failed on new data. By artificially expanding the dataset with transformations, the model encountered a wider variety of possible input scenarios, making it more adaptable to different environmental conditions such as lighting variations, dust accumulation, or weather effects like snow and cloud cover.

#### 3.5.4. Model Fine-Tuning

The system utilized the pre-trained VGG16 model [45], a deep convolutional neural network originally designed for large-scale image classification tasks [46], and fine-tuned it specifically for the solar panel dataset [47].The VGG16 architecture was selected for its simplicity, effectiveness, and suitability for the specific requirements of solar panel anomaly detection. While newer models such as ResNet and EfficientNet have demonstrated superior performance in various tasks, VGG16 was chosen due to its straightforward architecture when fine-tuning a pre-trained model for fault detection in photovoltaic systems. VGG16 has proven to be highly effective in image classification tasks, making it a reliable model for detecting anomalies in solar panels based on image data. Furthermore, VGG16 performs well with transfer learning, which allowed us to leverage pre-trained weights on large datasets, thus enhancing the model’s ability to generalize to smaller, domain-specific datasets, like those used in this study. The use of transfer learning with VGG16 provided an efficient means of improving model performance without requiring extensive computational resources or large amounts of labeled data.

The decision to use VGG16 was grounded in its proven track record, simplicity, and adaptability to the specific goals of this research, ensuring a reliable and efficient solution for the task of solar panel anomaly detection.

During this process, the earlier layers of the network responsible for detecting low-level features such as edges, textures, and shapes were kept frozen, as they are generally sufficient to be effective across different domains. However, the last few layers, which capture high-level features and make final predictions, were unfrozen and retrained on the solar panel dataset. By retraining these high-level layers, the model adapts to domain-specific features such as physical defects, environmental conditions, or anomalies like dust, cracks, or shading, which are critical for accurate fault detection in solar panels. This fine-tuning significantly improved the model’s ability to identify these specialized features, increasing its accuracy and robustness in detecting anomalies. This approach is especially beneficial because it combines the power of transfer learning with domain adaptation, enabling the system to efficiently learn from a relatively small dataset, while leveraging the general knowledge acquired from large-scale pre-training on broader image data.

#### 3.5.5. Classification Phase

**Figure 6:**
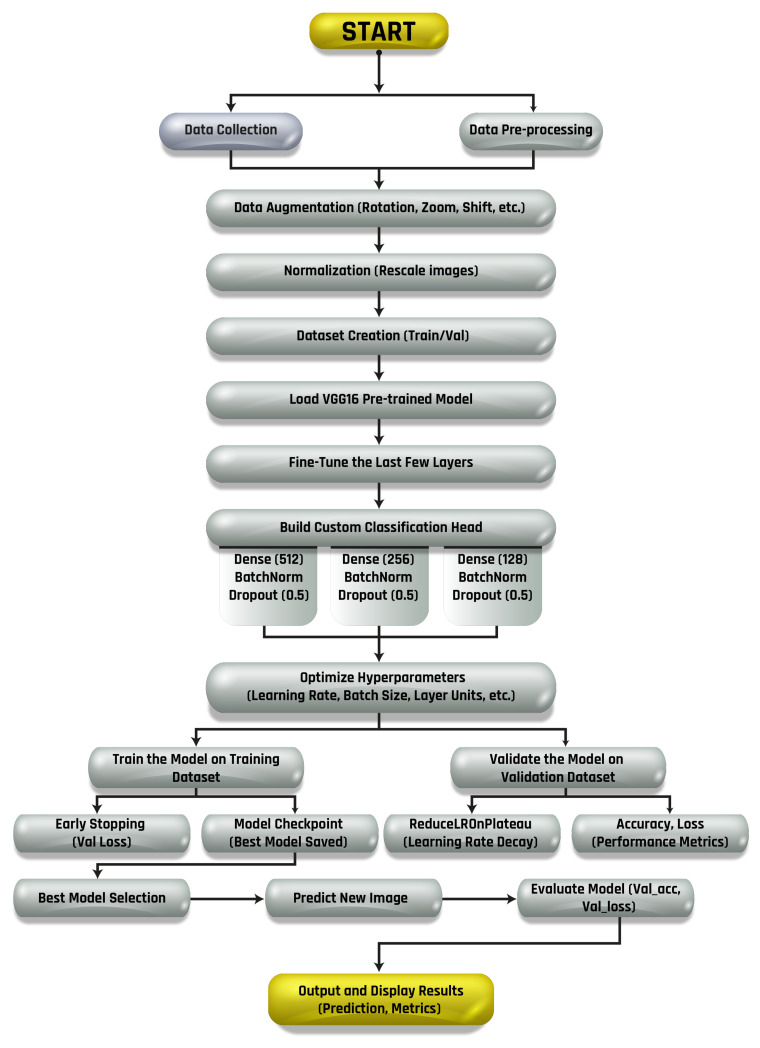
Methodology for the proposed architecture.

The model utilizes CNN layers to classify predictor variables. CNNs have proven effective in various energy-related applications due to their ability to predict outcomes, irrespective of the underlying probability distributions of the different labels. Their parallel architecture and learning capabilities enhance their efficiency in pattern classification, allowing them to effectively categorize observations into distinct classes. While these networks may have a lower fault tolerance, their research has shown that they can approximate any arbitrary function by adjusting the number of hidden layers and their associated parameters. The structure of the proposed deep neural network is detailed in Table 4 and illustrated in Figure 6.

#### 3.5.6. Real-Time Predictions

Once the model has been trained, it can be used to classify new images of solar panels in real time. This provides fast and accurate anomaly predictions, enabling quick responses to detected issues. By utilizing deep learning, this system can assist engineers and technicians in rapidly identifying faulty solar panels, enabling timely repairs and maintenance. The deep learning-based approach improves the operational efficiency by reducing the need for manual inspections, potentially lowering maintenance costs and minimizing downtime for solar energy systems.

### 3.6. Technologies for Model Implementation

#### 3.6.1. Tensorflow and Keras

The entire model was built using TensorFlow and Keras [48], two of the most widely adopted libraries for machine learning and deep learning.

- TensorFlow: This framework handles the complex mathematical operations of deep learning (e.g., gradient descent, backpropagation) and allows a model to execute efficiently on both GPUs and CPUs. TensorFlow also simplifies the training and deployment of neural networks, making it easy to integrate with various platforms.
- Keras: Keras acts as a high-level API over TensorFlow, providing an intuitive interface for defining layers, building models, and conducting experiments. In this project, Keras was used to define the architecture of the VGG16 model, as well as the custom layers added during fine-tuning.

#### 3.6.2. Vgg16 Pre-Trained Model

**Figure 7:**
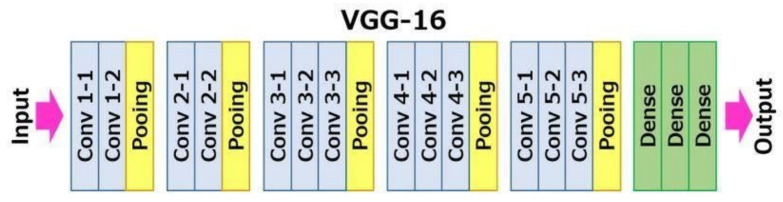
Architecture of VGG16.

VGG16 is a convolutional neural network (CNN) that is widely used for image classification tasks [49]. It was first introduced in 2014 by the Visual Geometry Group (VGG) at the University of Oxford. This deep network comprises 16 layers and has achieved state-of-the-art results on several benchmark datasets, including ImageNet, which contains over 14 million images spanning 1000 categories. VGG16 has proven to be highly effective due to its structure, shown in Figure 7, and its ability to generalize across various image classification problems. VGG16’s availability in deep learning frameworks such as Keras and TensorFlow further simplifies its use, offering easy implementation for machine learning tasks, especially when using pre-trained models through transfer learning.

#### 3.6.3. Transfer Learning

**Figure 8:**
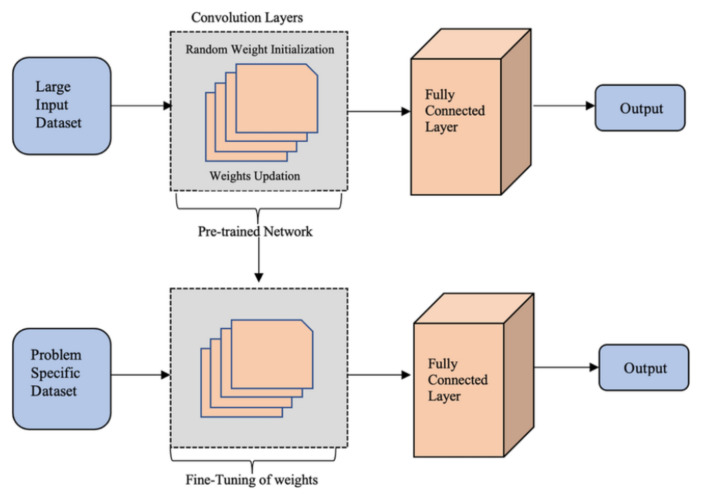
Architecture of transfer learning.

Transfer learning [50] is a technique where a model trained on one task is reused for another, related task, as depicted in Figure 8. This is particularly useful when limited data are available for the second task. In this paper, transfer learning was applied to leverage the power of VGG16, which was pre-trained on ImageNet, to detect anomalies in solar panels. The process of transfer learning with VGG16 started by loading the pre-trained model from the Keras library. The weights of the initial layers were frozen, allowing the model to retain the general features it had learned from ImageNet (such as edges, textures, and shapes). The final layers were then trained on our specific solar panel dataset, allowing the model to fine-tune itself for the task of detecting faults or anomalies in solar panels.

#### 3.6.4. Fine-Tuning

Fine-tuning [35] the last few layers of VGG16 allowed the model to adapt its high-level feature recognition to the specific characteristics of solar panel anomalies. By unfreezing these layers, the model could learn features relevant to this task, such as cracks or dirt that impact solar panel efficiency. In this project, we specifically used transfer learning and fine-tuning on VGG16 to build an efficient model for detecting solar panel anomalies. The combination of VGG16’s robust pre-trained features and our specific dataset led to improved accuracy and reduced the need for extensive data collection. Overall, VGG16 combined with transfer learning provides a powerful framework for image classification tasks, offering both high accuracy and ease of use, particularly when datasets are small or noisy. It is an effective tool for the rapid deployment of machine learning models in real-world applications such as solar panel fault detection.

The dataset, code, and developed application utilized for this research are publicly accessible, to enhance reproducibility, transparency, and accessibility for future studies in the domain of photovoltaic system fault detection. The dataset comprises labeled images of solar panels under various conditions, including classes for clean, dusty, physically damaged, electrically damaged, bird-dropping-covered, and snow-covered panels. This comprehensive dataset enables robust training and evaluation of the machine learning models employed.

The code repository, available on GitHub, includes the following components:

- **Data Preprocessing Scripts**: These scripts prepare the dataset by resizing, normalizing, and augmenting images to increase the robustness and prevent model overfitting. Key techniques include rotation, flipping, and zooming, to simulate real-world environmental conditions.
- **Model Training and Evaluation**: The repository contains the code for training the convolutional neural network (CNN) model, specifically leveraging a fine-tuned VGG16 architecture to classify anomalies in solar panels. The model is designed to identify faults with high accuracy, utilizing both transfer learning and fine-tuning to adapt to the specific characteristics of the solar panel dataset.
- **PyQt5 Application Interface**: A user-friendly interface developed with PyQt5 allows users to seamlessly interact with the model. This application simplifies the process of uploading images, viewing predictions, and understanding results, making it accessible to non-technical users as well.
- **Explainability Features**: The repository also includes code for implementing local interpretable model-agnostic explanations (LIME), an explainable AI technique. This feature helps users understand the factors influencing the model’s predictions, fostering trust and interpretability in AI-driven fault detection.

The link to the GitHub repository is provided in the **Data Availability Statement** section of this paper, ensuring that researchers and practitioners can replicate, validate, and build upon the methodologies developed in this study.

## 4. Results and Discussion

The following sections discuss in detail the data collection of environmental faults in solar panels and provide a comparative analysis of the trained and tested fault images. A PC with 16 GB RAM and a core i7 8th generation processor with Nvidia GPU was used, and the experimental configuration was created using Google Colabs and TensorFlow 2.4.0. A total of 889 real images of solar panels under various fault circumstances were taken. The images were subsequently separated into 80 and 20 percent groups for the training and testing of several neural networks using images of solar panels.

### 4.1. Performance Evaluation

Confusion matrixes are a valuable tool for evaluating the performance of machine learning models, enabling the assessment of metrics such as the AUC–ROC curve, recall, precision, and accuracy. They systematically assign predictions to the original classes of the data, helping to identify the classification accuracy for each record and highlighting potential areas of concern. In the matrix, the rows represent the actual labels from the training dataset, while the columns reflect the predicted outcomes of the model. This visualization aids in diagnosing the model’s effectiveness in accurately categorizing data.

A confusion matrix is a valuable tool for evaluating the performance of algorithms that classify outputs into binary categories, such as positive or negative (yes or no). It comprises four cells, each representing a unique combination of expected and actual outcomes. These cells help identify true positives (TP), true negatives (TN), false positives (FP), and false negatives (FN), allowing for a comprehensive analysis of a model’s accuracy and effectiveness in classification tasks. The following four outcomes are possible:

- True Positive (TP): This means that a prediction was correct. This is occasionally described as sensitivity.
- True Negative (TN): This denotes a negative prediction that materialized. This quality is known as specificity.
- False Positive (FP): Although the value was predicted to be positive, it turned out to be negative. Type-I errors are frequently used to describe this.
- False Negative (FN): The actual number was positive despite the negative forecast. A Type-II mistake is another name for this.

The expressions for all the statistical parameters are given as follows:

(1) $$\mathrm{Precision}=\frac{\mathrm{TP}}{\mathrm{TP}+\mathrm{FP}}$$

(2) $$\mathrm{Recall}=\frac{\mathrm{TP}}{\mathrm{TP}+\mathrm{FN}}$$

(3) $$\mathrm{TNR}=\frac{\mathrm{TN}}{\mathrm{TN}+\mathrm{FP}}$$

(4) F1-Score=2×Pr×RePr+Re

### 4.2. Model Hyperparameter Setting

The parameters utilized during the training of the CNN are detailed in Table 5. The training process was executed with a 20% hold-out for validation, employing a mini-batch size of 16 and 100 epochs. The trained CNN was then tested, and the accuracy was computed to validate the algorithm’s effectiveness. In the training, the Adam optimization algorithm with a learning rate of 3e−4, Mini-Batch Size 16, L2 regularization with a factor of 0.0001, and piecewise learning rate schedule was used, with a drop factor of 0.9 every 3 epochs. The execution environment was the CPU.

### 4.3. Model Evaluation

In the feature extraction process using an encoder/decoder approach, the first layer consisted of an autoencoder with 2500 hidden nodes trained on the training dataset. The second layer employed another autoencoder, this time with 3400 hidden nodes, which were trained on the features obtained from the first layer. Subsequently, a SoftMax layer was trained on the features extracted from the second autoencoder. To enhance the efficiency, a dimensionality reduction procedure was applied to eliminate features that contributed the least to the predictive variable. Retaining these irrelevant features could have negatively impacted the model’s overall performance.

**Figure 9:**
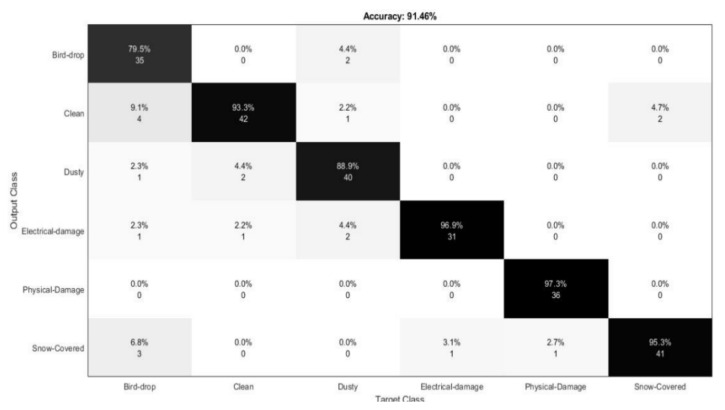
Correlation matrix of the solar panel dataset for the proposed model.

A thorough analysis of the confusion matrix presented in Figure 9 reveals that some key misclassifications occurred between categories such as physical damage, cleaning, and dust. These misclassifications may have arisen due to the similarities in the visual features of these categories, particularly when certain types of faults like physical damage appeared visually similar to dust accumulation or dirt on the surface of the solar panel. To better understand these misclassifications, we analyzed the correlation matrix heatmap of the dataset, which visually illustrates the relationships between features. The heatmap highlights which features were most strongly correlated with each other. High correlations between features can introduce redundancy, which may affect the stability of the model and its ability to distinguish between similar categories. The proposed model achieved an accuracy of 91.46%, reflecting its overall effectiveness, but also indicating areas for improvement in distinguishing between visually similar faults.

### 4.4. Xai (LIME) Feature Importance

LIME evaluates the local fidelity of a model, which ensures that it effectively captured characteristics relevant to the predictions made. While local fidelity aims to describe a prediction’s context, it may not always align perfectly with the global behavior of a model. LIME analyzes the immediate surroundings of a prediction to assess its local accuracy and provide an explanation. For instance, if a prediction is accurate but not aligned with the global model, LIME seeks high probability features in that vicinity to clarify the model’s decision-making process.

### 4.5. Pyqt5 Implementation

**Figure 10:**
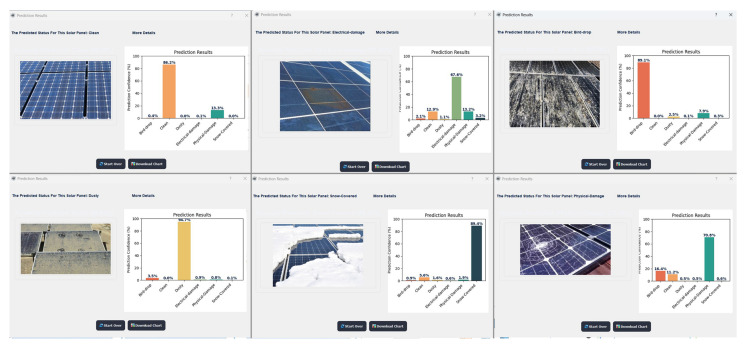
Results with PyQt5 implementation.

The results obtained from the PyQt5 interface demonstrate the high accuracy of the developed model in detecting anomalies in photovoltaic (PV) panels, as shown in Figure 10. Built with PyQt5, the user interface provides a practical and user-friendly platform for real-time interaction with the fault detection system. It allows users to upload images of PV panels and receive immediate diagnostic feedback, displaying predictions directly on-screen, along with visual indicators of the identified faults.

The high accuracy achieved by the model, as illustrated in this figure, demonstrates the effectiveness of combining CNN architecture with fine-tuning on a solar panel-specific dataset. The fine-tuning of the VGG16 CNN model enabled it to learn the unique features associated with common PV panel faults, such as dust accumulation, physical damage, and electrical anomalies, which are critical for maintaining an optimal solar energy output.

This interactive application facilitates the timely identification and diagnosis of faults, reducing the need for manual inspection and contributing to improved operational efficiency and reduced downtime in solar energy systems.

**Figure 11:**
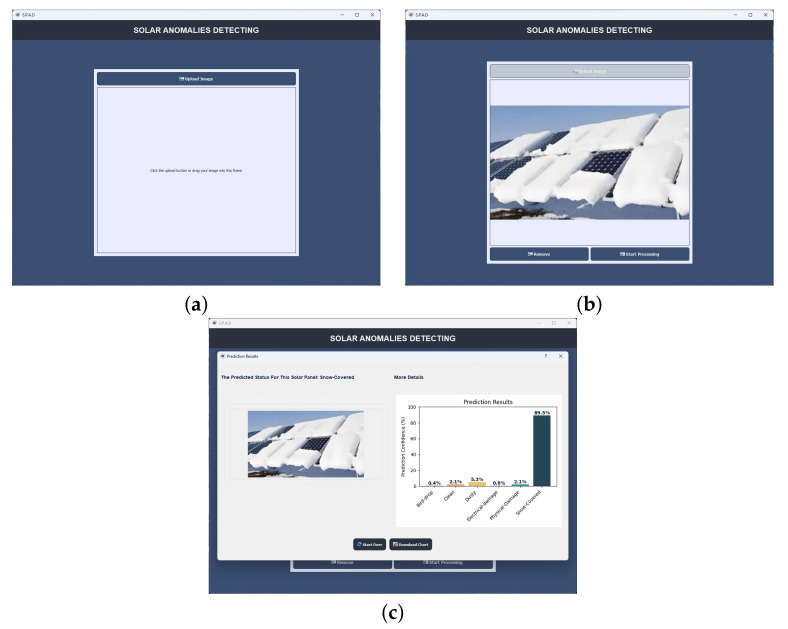
SPAD upload image (**a**), image preview (**b**), and prediction result implementation (**c**).

Generating a report from the application involves three steps, as shown in Figure 11. First, the user is prompted to upload an image of a solar panel that they wish to analyze for potential anomalies, using the **Upload Button**. This can be achieved by selecting an image file from the device or by simply dragging and dropping it into the designated area of the application interface. Once the image has been uploaded, a **Preview Screen** displays the image, allowing the user to confirm or remove it if needed. At this stage, the user can proceed by selecting the *Start Processing* button, which initiates the anomaly detection analysis of the uploaded image.

After processing, the application moves to the **Prediction Results Screen**, where the detected anomaly type, such as “Dusty”, is displayed along with a confidence level (e.g., 94.9%). Additionally, a detailed chart visualizes the model’s confidence levels across various potential anomalies, such as ”Bird-drop”, “Dusty”, and “Physical-Damage”. Users can download this chart for documentation purposes by selecting the *Download Chart* button. Finally, to begin a new analysis, users can reset the application using the *Start Over* button, which clears previous data and returns the interface to the initial upload screen.

## 5. Conclusions and Future Works

Developing technologies to control solar panel energy generation has proven essential for higher reliability and lower costs. As a renewable energy source, solar panels provide power without releasing any pollution. However, dirt, a significant environmental element impacting energy generation, negatively affects the performance of solar panels. When dirt builds up on the surface of a solar panel, the amount of light that strikes it is diminished, thereby reducing the panel’s ability to produce electrical energy. This paper successfully implemented a deep-learning model to classify solar panel anomalies by fine-tuning the VGG16 architecture. By leveraging pre-trained models, extensive data augmentation, and powerful optimization techniques such as the Adam optimizer, the model can accurately predict anomalies in solar panels based on image data. The model performed well through the effective application of transfer learning and data augmentation, achieving better validation performance and reducing overfitting.

The reported test accuracy underscores the potential of this implementation in real-world scenarios. The proposed model achieved a 91.46% accuracy, specificity of 98.29%, and F1 score of 91.67%. The model could properly classify various fault sources, demonstrating its effectiveness in practical applications.

While the model showed robust performance in anomaly detection, it is important to note some limitations. The model’s performance heavily depends on the quality and diversity of the training data. In cases where images are of low resolution or under suboptimal lighting conditions, the detection accuracy could be affected. Despite these limitations, the model’s robustness, particularly in the classification of dirt-related issues, positions it as a valuable tool for real-time monitoring of solar fields. In practice, it can help optimize maintenance schedules, reduce energy losses due to panel inefficiency, and extend the lifespan of solar installations.

In light of this study’s findings, several potential areas for future research and practical application have been identified. Future research should focus on evaluating the long-term effects of dust accumulation on photovoltaic performance, exploring how variations in dust type, density, and particle size influence energy output. Furthermore, we envision integrating video input, such as drone-captured footage, into the system to enable real-time status detection of solar fields. This addition would enhance the monitoring capabilities of the application, providing dynamic, in situ assessments of panel conditions and further expanding the practical applications of this study.

## Figures and Tables

**Table 1:** Review of computer vision (CV) applications in various fields.

Reference	Year	Application
[18]	2024	This paper introduced the RDA-MTE deep learning model for emotion recognition, integrating real-time emotion analysis with sports behavior decision-making.
[19]	2023	This survey explored the role of CV in intelligent transportation systems (ITS), highlighting its applications in traffic monitoring, incident detection, and road condition monitoring.
[20]	2020	This paper provided an overview of CV-based indoor localization methods, classifying them based on configuration stage and sensing devices.
[21]	2020	This paper surveyed the use of CV and ambient intelligence for healthcare, with a particular focus on children’s health.
[22]	2021	This survey compared various CNN architectures, discussing their strengths, weaknesses, applications, and future research directions.

**Table 2:** Summary of prior research in solar panel fault detection.

Reference	Year	Technique
[27]	2019	Region-based CNN with a recall rate over 90% and a false positive rate around 2–3%, tested on a dataset of nearly 9000 solar panels.
[28]	2022	Defective PV module region object detection using the Res-CNN3 framework.
[29]	2020	UNet, FPNet, and LinkNet are examples of deep neural networks (DNNs). The accuracy of this work was 89.63%.
[30]	2020	The suggested approach, which uses a fine-tuned pre-trained CNN, performed better than current methods and achieved a high fault detection accuracy of 73.53%. The accuracy achieved was 73.53%.
[31]	2021	A CNN correctly categorized a range of issues using photos taken by unmanned aerial aircraft (UAVs). It had an accuracy of 95.07%.
[32]	2021	Convolutional neural network (CNN) and chaos synchronization detection method (CSDM) hybrid algorithm for PV module failure detection research. The accuracy achieved was 86.75%.
[33]	2021	One-dimension convolutional neural networks (1-D CNN) and multilayer perceptrons (MLP) are examples of deep neural networks. It had a rate of 89.75%.

**Table 3:** Description of solar panel dataset.

Class	Bird-Drop	Clean	Dusty	Electrical Damage	Physical Damage	Snow Covered
Num of Images	206	194	191	104	70	124

**Table 4:** Description of CNN architecture.

Input Layer	
Feature Input Layer:	This is the entry point of the data into the neural network.
numFeatures:	The number of input features that the network expects. It is essential that the input data match this dimensionality.
Normalization:	The ’zscore’ argument indicates that the input data will be normalized by subtracting the mean and dividing by the standard deviation. This normalization can help in speeding up training and improving convergence.
**First Hidden Layer Block**	
Fully Connected Layer (1500 Neurons):	This dense layer has 1500 neurons and will learn from the input features.
Batch Normalization Layer:	Normalizes the activations of the neurons, helping improve the training speed and stability of the network.
Leaky Rectified Linear Unit (Leaky Relu) Activation Function:	This function allows small negative values when the neuron is not active. It can sometimes prevent “dead neurons” in a network.
Dropout Layer (60%):	Randomly sets 60% of the layer’s outputs to zero during training to prevent overfitting.
**Second Hidden Layer Block**	
Fully Connected Layer:	1000 neurons in the fully connected layer.
Relu Activation Function:	Standard Relu activation function, which sets all negative values to zero.
Dropout Layer:	50% dropout rate.
**Third Hidden Layer Block**	
Fully Connected Layer:	500 neurons in the dense layer.
Leaky Relu Activation Function:	Leaky Relu activation.
Dropout Layer:	40% dropout rate
**Fourth Hidden Layer Block**	
Fully Connected Layer:	250 neurons in the fully connected layer.
Relu Activation Function:	Standard Relu activation function, which sets all negative values to zero.
Dropout Layer:	40% dropout rate.
**Output Layer**	
Fully Connected Layer:	This has a neuron for each class in the classification task. If there are 10 classes, numClasses would be 10, and there would be 10 neurons.
SoftMax Layer:	Converts the output of the previous layer into probability scores for each class.
Classification Layer:	Determines the final output class based on the probabilities from the SoftMax layer

**Table 5:** Parameter values for CNN during training.

Parameter	Value
Solar Panel	
Optimizer:	Adam
Learning Rate:	0.0001
Loss Function:	Cross entropy
Metrics:	Accuracy
Batch Size:	15
Epochs:	100

## Data Availability

This article uses private data that are made available through a GitHub repository. The dataset and the code used for the machine learning comparison in this study can be accessed at the following location: https://github.com/Ledmaoui/Solar-Panel-Anomalies-Detecting link (accessed on 1 January 2024).

## Abbreviations

The following abbreviations are used in this manuscript:

AI	Artificial Intelligence
ANN	Artificial Neural Network
AUC-ROC	Area Under the Receiver Operating Characteristic Curve
CC BY	Creative Commons Attribution
CNN	Convolutional Neural Network
CSDM	Chaos Synchronization Detection Method
CV	Computer Vision
DIY	D.I. Yogyakarta
DL	Deep Learning
FN	False Negative
FP	False Positive
FPNet	Feature Pyramid Network
GHGs	Greenhouse Gases
GW	Gigawatt
IoT	Internet of Things
IEA	International Energy Agency
kNN	k-Nearest Neighbors
LIME	Local Interpretable Model-Agnostic Explanations
LSTM	Long Short-Term Memory
ML	Machine Learning
MAE	Mean Absolute Error
MLP	Multilayer Perceptron
PV	Photovoltaic
PVS	Photovoltaic System
ReLU	Rectified Linear Unit
SHAP	Shapley Additive Explanations
SVM	Support Vector Machines
SPAD	Solar Panel Anomalies Detection
TN	True Negative
TP	True Positive
UNet	U-shaped Neural Network
UAV	Unmanned Aerial Vehicle
VGG	Visual Geometry Group
